# Non-viral siRNA delivery into the mouse retina *in vivo*

**DOI:** 10.1186/1471-2415-10-25

**Published:** 2010-10-01

**Authors:** Andrey Turchinovich, Georg Zoidl, Rolf Dermietzel

**Affiliations:** 1 Department of Neuroanatomy and Molecular Brain Research, Ruhr-University Bochum, Bochum, Germany; 2 Department of Cytology, Ruhr-University Bochum, Bochum, Germany; 3 International Graduate School of Neuroscience, Ruhr-University Bochum, Bochum, Germany

## Abstract

**Background:**

Gene silencing in the retina using RNA interference could open broad possibilities for functional studies of genes *in vivo *and for therapeutic interventions in eye disorders. Therefore, there is a considerable demand for protocols to deliver siRNA into the vertebrate retina. In this work we explored a possibility to deliver synthetic 21 bp siRNA into the mouse retina after intravitreal application using a non-viral carrier.

**Methods:**

Fluorescently labelled synthetic 21 bp siRNA duplex was combined with Transit-TKO transfection reagent and injected intravitreally into adult mice eyes. Eyes cryostat sections and whole mount retinas were prepared 24-48 h post-injection, stained with either Hoechst 33342 (cell nuclei) or immunostained with anti-GFAP antibody (astroglia cells marker). Distribution of fluorescent siRNA signal in the retina was investigated.

**Results:**

Single intravitreal injection of as little as 5 ng of siRNA combined with Transit-TKO transfection reagent by a modified protocol provided robust and non-toxic delivery of the siRNA into the retina. However, siRNA accumulation was predominantly confined to ganglion cells layer as analysed 24 h post-injection. Furthermore, siRNA containing particles were localized along GFAP cytoskeleton of retinal astroglial cells hinting on intracellular localization of the siRNA

**Conclusions:**

In this work we demonstrated that siRNA can be efficiently delivered into the vertebrate retina in vivo with low-toxicity using a non-viral carrier, specifically Transit-TKO transfection reagent. However, the capacity of siRNA delivered by our protocol to induce gene silencing in the retina has to be further evaluated. Our report could raise a closer look on Transit-TKO transfection reagent as a promising siRNA carrier in vivo and be of interest for the researchers and companies who work on development of ocular RNAi techniques.

## Background

The technology of RNA interference (RNAi) offers the perspective for selective and on demand silencing of gene expression. One of the critical factors that limit the experimental and therapeutic application of RNAi *in vivo *is the ability to deliver intact siRNA efficiently. Although RNAi technology has been successfully demonstrated for cell lines and primary cultures, delivery of siRNA in mammalian tissues *in vivo *provides a significant challenge [1]. Particular difficulties have been associated with non-viral gene transfer into the retina *in vivo *[2]. One of the challenges is to overcome the inner limiting membrane, which impedes the transfection of the retina [3,4]. Additionally, negatively charged sugars of the vitreous have been shown to interact with positive DNA-transfection reagent complexes promoting their aggregation, impeding diffusion and cellular uptake [5,6]. To date reports describing ocular siRNA applications are amounting. However, strategies are divergent and the outcome is highly variable. Some experiments showed successful knock-down after injection of naked unmodified or nuclease protected siRNA to a number of tissues including retina. Intravitreal application of "naked" stability modified siRNA directed against vascular endothelial growth factor (VEGF) or VEGF receptor 1 suppressed ocular neovascularisation in rats [7]. However, a phenomenon of sequence independent suppression of retinal neovascularisation by siRNAs has been recently reported by Kleinmann and colleagues [8]. Furthermore, some reports indicate that naked 21-nt siRNA can not be internalized into the cells unless cell-permeating moieties are used [9]. Moreover, besides poor cellular uptake, unprotected siRNA is prone to relatively rapid degradation in the vitreous [10]. A combination of siRNA with common commercial transfection reagents including Lipofectamine 2000 [11] and in vivo jetPEI [12] proved to be successful for shRNA encoding plasmid DNA delivery into the retina through intravitreal application. Transit-TKO transfection reagent has been previously used as a carrier to deliver siRNA subretinally [13] and subconjuctivally [14]. In this work, we have tested the feasibility of using Transit-TKO transfection reagent for siRNA delivery into the mouse retina after intravitreal injection.

## Methods

### Intravitreal injections

The intravitreal injection procedure had a proper permission of the Ruhr University Bochum according to German animal protection law. Wild-type mice of C57BL/6 strain (6 month old) were anesthetized by intraperitoneal injection of ketanest/xylazine (ketanest, 100-125 mg/kg; xylazine, 10-12.5 mg/kg). Intravitreal injections were performed under a dissecting microscope with a 32 gauge needle attached to a 5 μl glass syringe (Hamilton, Reno, USA). The needle was positioned 1 mm posterior to the limbus and 1 μl of the solution was slowly (3-5 seconds) injected into the vitreous chamber of the eye. A 20 second interval was kept before removing the needle.

### Preparation of Transit TKO-siRNA complexes

Transit-TKO transfection reagent was purchased from Mirus Bio (Madison, USA). For monitoring the transfection efficacy we used non-specific 21 base pairs long DY-547 labelled siRNA duplex (siGlo) provided by Dharmacon (Lafayette, USA). The optimised protocol was as follows: 0,1 μg (7,5 pmol) of siRNA was combined with 4,5 μl of Transit-TKO in 50 μl of H_2_O and incubated for 20 min at RT. Water was used as a solvent to minimize the size of the transfection particles and to prevent aggregates which may impair penetration through the inner limiting membrane into the retina. Afterwards, the solution was evaporated under vacuum and precipitate was dissolved in either 5 μl or 20 μl H_2_O shortly before injection.

### Retina cryostat sections

Mice were anesthetized by asphyxiation in CO_2 _and euthanized by cervical dislocation 24 h after intravitreal injection. The eyes were enucleated, covered in tissue freezing medium (Leica, Nussloch, Germany) and frozen in 8% methylcyclohexan in 2-methylbutan (v/v;-60°C). Sagittal cryosections, 12 μm thick, were prepared and placed on Superfrost Plus slides (Menzel-Glaser, Braunschweig, Germany). Sections were dried for 60 min at 40°C and fixed in 4% paraformaldehyde for 10 min. For staining cells nuclei cryosections were incubated in 1:10,000 of Hoechst 33342 dye (Promega, Madison, USA) for 20 min, rinsed in PBS and mounted with ProLong Antifade kit (Molecular Probes). Fluorescent images were analysed by fluorescent microscopy.

### Immunohistochemistry on whole mount retinas

Mice were anesthetized by asphyxiation in CO_2 _and euthanized by cervical dislocation 48 h after intravitreal injection. The eyes were enucleated; retinas were dissected under a binocular using fine forceps and fixed in 4% para-formaldehyde in PBS at 4°C for 2 hrs. Following fixation, retinas were rinsed in PBS and incubated in blocking solution containing 10% normal goat serum diluted in PBS with 1% Triton-X-100 for 2 hrs at room temperature. Afterwards retinas were incubated with mouse monoclonal anti-GFAP antibodies (1:100) (Sigma-Aldrich, St. Louis, USA) in PBS with 0,1% Tween 20 for 2 days at 4°C. Retinas were rinsed three times in PBS for 30 min at room temperature, followed by an overnight incubation at 4°C with secondary antibody (Alexa Fluor^® ^546 red goat anti-mouse, Molecular Probes, Leiden, Netherlands) at 1:1,000 dilution in PBS containing 2 mg/ml of bovine serum albumin. Retinas were washed 5 times for 30 min each in PBS and mounted (ganglion cell layer side up) with ProLong Antifade kit (Molecular Probes). The specimen were analysed by confocal microscopy (Zeiss, LSM 510 Meta).

## Results

In this work we explored Transit-TKO transfection reagent as a carrier to deliver siRNA into the mouse retina in vivo through intravitreal injection. As a base protocol for Transit-TKO/siRNA formulation we used the manufacturer's instructions for in vitro transfection. However, we further modified siRNA complexation procedure in a way to reduce the average size of the siRNA-reagent complexes, therefore increasing their mobility in the vitreous. For that purpose we 1) combined siRNA with Transit-TKO transfection reagent in water (instead of serum free media) in order to reduce the ionic strength of the solution; 2) performed complexation of siRNA with the reagent initially in a diluted volume (50 μl) with subsequent concentration by speed vacuum evaporation. The concentration of the siRNA/Transit-TKO solution was required due to the fact that the maximum volume of 1 μl could be injected into the mouse vitreous at once.

**Figure 1:**
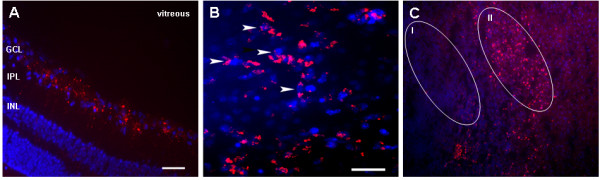
**Intravitreal injection of siRNA**. **A**. Fluorescent micrograph of a cryostat section and **B), C) **whole mount retinas prepared 24 h after single intravitreal injection with 20 ng fluorescent siRNA (red) combined with Transit-TKO in low ionic environment. Note, the prominent localization of siRNA in the ganglion cells layer (GCL) of the retina. Arrows indicate the examples of cytoplasmic accumulation of siRNA near to cells nuclei. Low and highly transfected regions of the retina are marked by ovals (I, II). Cells nuclei were counterstained with Hoechst (blue). Scale bar = 20 μm.

After single intravitreal injection of as little as 20 ng of siRNA combined with Transit-TKO reagent robust transfection of the retina was observed. Fluorescent signals of siRNA were reproducibly detected in the ganglion cells layer (GCL) of the retina as aggregates of variable sizes 24 h post-injection (Fig. 1A). Significantly fewer aggregates were found in the inner plexiform and inner nuclear layers suggesting that siRNA uptake was limited to the innermost cell layer 24 h post injection. However, we did not analyze the dynamic of siRNA penetration into the retina in later time points. In sections of whole mount retina preparations a typical distribution of siRNA in the ganglion cells layer with fluorescent particles frequently located close to the cell nuclei was observed (see arrows; Fig. 1B). However, siRNA accumulation was not uniform along the retina, revealing regions of high and low transfection (Fig. 1C indicated by oval regions).

**Figure 2:**
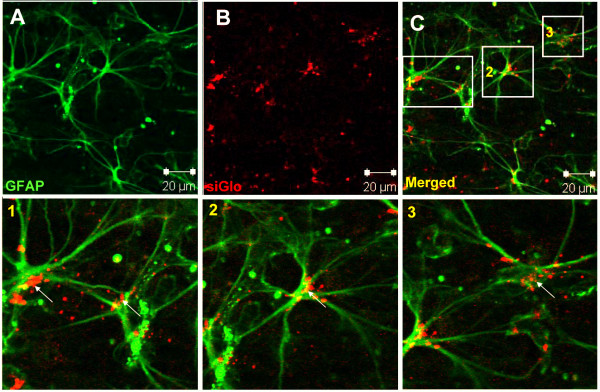
**Localisation of siRNA in retinal astrocytes**. Confocal micrographs obtained after anti-GFAP (green) immunostaining of whole mount retinas performed 48 h after single intravitreal injection of 5 ng fluorescently labelled siRNA (siGlo, red) combined with Transit-TKO. The pictures in A-C show a single plane through the vitreal surface of the retina highlighting prominent sides of siRNA localization. **A **- GFAP; **B **- siRNA (siGlo); **C **- Merged. The localization of siRNA along GFAP processes and cell bodies is indicated by arrows (enlarged in frames 1, 2, 3)

Next, we investigated intracellular localization of siRNA in the retinal astrocytes which reside on the boundary of ganglion cells layer and the vitreous. Whole mount retinas prepared 48 h after single intravitreal injection of 5 ng siRNA/Transit-TKO were stained for astrocytes specific glial fibrillary acidic protein (GFAP) (Fig. 2). siRNA particles were localized within or along the astrocytes cell bodies and processes hinting on their intracellular localization (Fig. 2 indicated by arrows). We did not measure the damage of the retina after intravitreal injection of 20 ng of siRNA. However, no alterations in GFAP cytoskeleton was observed when using 5 ng of siRNA per injection suggesting that retinal astrocytes were not impaired by the treatment.

## Discussion

We explored a number of possibilities to deliver synthetic 21 bp long siRNA duplex into the mouse retina after single intravitreal injection. We have found, however, that injecting up to 5 μg of naked fluorescently labelled siRNA into the vitreous did not provide any visible siRNA accumulation in the retina (as analysed 24 h post-injection). This can be explained by rapid degradation of unprotected siRNA in the vitreous. Mixing siRNA with common cationic lipids-based transfection reagents including siLentFect (BioRad) and Oligofectamine (Invitrogen) in different siRNA/reagent proportions also did not induce any visible siRNA uptake by retinal cells after intravitreal injection (data not shown). However, both of these transfection reagents provided robust penetration of siRNA into mammalian cells in culture. These results were consistent with previous reports indicating that the efficient transfection of the retina is difficult to achieve [2-6]. Among the difficulties are the inner limiting membrane that separates vitreous cavity from ganglion cells layer and the vitreal fluid that constitute a non-permissive environment for the transfection of the retina with cationic lipids.

Transit-TKO transfection reagent mixtures with siRNA has been previously reported to successfully knockdown genes in pulmonary tissue after intranasal delivery [15], retinal pigmented epithelium after subretinal injection [13] and corneal fibroblasts after subconjuctival injection [14]. This motivated us to test this approach to deliver siRNA through intravitreal application - more challenging and at the same time less invasive route for the retina transfection. Our modified protocol mediated robust delivery of the siRNA through the inner limiting membrane into the retina. Furthermore, siRNA containing particles colocolized (or resided in a close proximity) with astrocytic GFAP cytoskeleton and cells nuclei indicating intracellular localisation.

We had also tested the ability of siRNA delivered by our protocol to induce silencing of the gap junction protein Cx43. Cx43 is highly expressed by retinal astrocytes - the layer that resides in the vitreal surface of the retina. However, analysis of Cx43 mRNA level performed by qRT-PCR on whole dissected retinas did not reveal any detectable Cx43 knockdown 96 h after injection of Cx43 directed siRNA (data not shown). It is important to mention, that in our hands the area of the retina containing fluorescent signals of siRNA varied significantly from injection to injection and was not greater than 30% (usually 10-30% as analyzed by subjective assessment of at least N = 12 eyes). Therefore it remains unclear whether the absence of Cx43 knockdown in our tests can be attributed to the inability of siRNA delivered by our formulation protocol to mediate functional effect in the retina. We assume that our protocol has still too preliminary efficacy to assess siRNA functionality. We performed intravitreal injection manually, without a help of a stereotactic device. Although this approach is valuable for fast screening of a large number of different siRNA/reagent mixtures, more delicate control of intravitreal injections could require achieving uniform and complete retina transfection. Thus, elimination and distribution of compounds in the vitreous have been previously described [16,17]. In rodents, the procedure for intravitreal injection is additionally complicated by the large lens size and relatively small vitreous cavity. Therefore, extraocular loss of injected solution can be significant and increases for higher injected volumes, with larger standard deviations [17].

## Conclusions

In this work we developed a modified protocol for the formulation of siRNA with Transit-TKO transfection reagent. siRNA combined by our protocol was capable to penetrate through the inner limiting membrane into the retina and accumulated in ganglion cells layer including retinal astrocytes. However, more delicate control of the conditions for intravitreal injections is needed to reduce treatment variability and to increase uniformity of transfection. Nevertheless, our formulation protocol can serve as a starting point for developing more uniform and complete siRNA transfection of the retina in vivo. Our report could raise a closer look on non-viral carriers for intraocular siRNA delivery and be of interest for the researchers and companies who work on development of ocular RNAi techniques.

## Competing interests

The authors declare that they have no competing interests.

## Authors' contributions

All authors read and approved the final manuscript. AT designed and curried out experimental part and wrote the manuscript; GZ participated in the design of retinas cryosectioning and whole-mount retina immunostaining and revised the manuscript; RD conceived of the study, participated in its design and coordination and revised the manuscript. All authors read and approved the final draft.

## Pre-publication history

The pre-publication history for this paper can be accessed here:

http://www.biomedcentral.com/1471-2415/10/25/prepub
